# Comments on “Network Pharmacology‐Based and Experimental Validation Elucidate the Target Mechanism of Vinorine in Ameliorating Secondary Brain Injury After Intracerebral Hemorrhage”

**DOI:** 10.1002/cns.70722

**Published:** 2025-12-23

**Authors:** Xihan Ying, Jieqi Zhang, Ruijie Ma

**Affiliations:** ^1^ Key Laboratory of Acupuncture and Neurology of Zhejiang Province, The Third School of Clinical Medicine (School of Rehabilitation Medicine) Zhejiang Chinese Medical University Hangzhou China; ^2^ Department of Acupuncture The Third Affiliated Hospital of Zhejiang Chinese Medical University Hangzhou China

Dear Editor,

We have carefully reviewed the article titled “Network Pharmacology‐Based and Experimental Validation Elucidate the Target Mechanism of Vinorine in Ameliorating Secondary Brain Injury After Intracerebral Hemorrhage” by Wu et al. [1].

Through a combination of network pharmacology and in vitro/in vivo experiments, the authors propose that Vinorine (Vin) mitigates secondary damage following intracerebral hemorrhage by modulating the CXCR2–JAK–STAT pathway, downregulating MMP expression, and promoting neuronal survival. This study provides important clues regarding the potential of natural products in treating cerebral hemorrhage. We would like to offer some suggestions to further enhance the reliability and completeness of the research.

Firstly, intraperitoneal (ip) administration was employed in this study. While this route facilitates animal experimentation, the drug must first enter the central nervous system via the peripheral circulation. Peripheral metabolism and immune regulation may influence the assessment of direct central effects. In contrast, intrathecal, intracerebroventricular, or intrastriatal injection allows for more direct targeting of the lesion site [2, 3], thereby better facilitating the validation of Vin's central target effects; or intravenous injection, which is commonly used in clinical practice, also has certain advantages in terms of drug distribution and metabolism, and can provide a reference that is closer to clinical reality for the study. However, regardless of the new injection method used, appropriate toxicological validation should be conducted to ensure its safety and efficacy. Secondly, the authors observed that Vin can restore the integrity of the blood–brain barrier. CXCR2 is primarily expressed on neutrophils [4], which are known to disrupt the integrity of the blood–brain barrier through infiltration [5]. Although studies have shown that CXCR2 expression also increases during the activation of microglia [6], the authors did not perform immunofluorescence colocalization of CXCR2 with either microglia or neutrophils. Therefore, the reduction of CXCR2 expression by Vin may be achieved through the decrease of neutrophil infiltration. We propose that this effect may offer an alternative mechanism: BBB repair reduces the infiltration of peripheral immune cells—such as neutrophils—into brain parenchyma, thereby alleviating local inflammation [7]. This suggests that Vin's neuroprotective effects may not solely depend on regulating the JAK–STAT pathway in microglia, but also involve inhibiting the invasion of peripheral immune cells.

Therefore, subsequent studies may consider combining single‐cell transcriptome sequencing with flow cytometry. By comparing the Vin‐treated group with the vehicle control group, it could be interesting to analyze whether the ratio of neutrophils changes and to explore the potential effects of Vin on the transcription of microglia. Additionally, attempting co‐staining for CXCR2, neutrophil markers, and microglial markers in vivo might help further investigate whether Vin's neuroprotective effects are associated with direct actions on microglia. Furthermore, early intervention is critically important for patients with intracerebral hemorrhage in clinical settings [8]. The study employed a 24‐h dosing time point; it is recommended that future research explore different time points within 24 h post‐modeling, such as at 6, 12, and 24 h after the onset of intracerebral hemorrhage, to investigate the effects of intervention at different time points. This multi‐time‐point experimental design will help determine the optimal therapeutic window and provide stronger scientific evidence for early clinical intervention.

In summary, the work by Wu et al. is of significant importance. We believe that further addressing the aforementioned issues will contribute to a more comprehensive understanding of Vin's mechanism of action and enhance its clinical translational value.

## Conflicts of Interest

The authors declare no conflicts of interest.

## Data Availability

Data sharing is not applicable to this article as no datasets were generated or analyzed during the current study.
